# The BK Activator NS11021 Partially Protects Rat Kidneys from Cold Storage and Transplantation -Induced Mitochondrial and Renal Injury

**DOI:** 10.1016/j.abb.2020.108410

**Published:** 2020-05-21

**Authors:** Stephen Shrum, Julia Tobacyk, Sorena Lo, Nirmala Parajuli, Lee Ann MacMillan-Crow

**Affiliations:** 1 Department of Pharmacology and Toxicology, College of Medicine, University of Arkansas for Medical Sciences, 4301 W. Markham Street, Mail Slot 611, Little Rock, AR 72205; 2 Arkansas Children’s Research Institute, 1 Children’s Way, Little Rock, AR 72202

**Keywords:** mitochondria, kidney transplantation, cold storage, oxidative stress, mitoBK channel, NS11021

## Abstract

Kidneys from deceased donors used for transplantation are placed in cold storage (CS) solution during the search for a matched recipient. However, CS induces mitochondrial and cellular injury, which exacerbates renal graft dysfunction, highlighting the need for therapeutic interventions. Using an *in vitro* model of renal CS, we recently reported that pharmacological activation of the mitochondrial BK channel (mitoBK) during CS protected against CS-induced mitochondrial injury and cell death. Here, we used an *in vivo* syngeneic rat model of renal CS (18 hr) followed by transplantation (24 hr reperfusion) (CS+Tx) to similarly evaluate whether addition of a mitoBK activator to the CS solution can alleviate CS+Tx-induced renal injury. Western blots detected the pore-forming α subunit of the BK channel in mitochondrial fractions from rat kidneys, and mitoBK protein expression was reduced after CS+Tx compared to sham surgery. The addition of the BK activator NS11021 (3 μM) to the CS solution partially protected against CS+Tx-induced mitochondrial respiratory dysfunction, oxidative protein nitration, and cell death, but not acute renal dysfunction (SCr and BUN). In summary, the current preclinical study shows that pharmacologically targeting mitoBK channels during CS may be a promising therapeutic intervention to prevent CS+Tx-induced mitochondrial and renal injury.

## 1. Introduction

The vast majority of donor kidneys (70% in the US) provided by deceased donors undergo cold storage (CS) preservation prior to transplantation (2018 UNOS website data). Cold storage mediated organ preservation was developed in the early 1980s by the groundbreaking research of Belzer and Southard [1–4]. The CS solution is designed to hypothermically mitigate ischemic injury to donor kidneys by slowing the renal metabolic rate, and the clinical objective of the CS process is to extend the window of time available to find optimally matched recipients (reviewed by Salahudeen et al [5]). Ultimately, the advent of CS has greatly expanded the number of available donor kidneys, and CS is considered to be a clinically vital procedure. However, CS induces renal injury, exacerbates renal dysfunction, and greatly increases the chance of transplant failure (5-fold) compared to transplanted kidneys that have not undergone CS [5–7]. Unfortunately, there are few clinical interventions to protect donor kidneys from CS-induced damage, which is the focus of the current study.

Our laboratory and others have shown that mitochondrial injury is a key triggering event of CS-induced renal injury [8–13]. Using renal tubular cell lines exposed to CS followed by rewarming (CS+RW), and employing clinically relevant rat models of renal CS followed by transplantation (CS+Tx), numerous studies have revealed that CS induces respiratory complex dysfunction, mitochondrial reactive oxygen species (ROS), mitochondrial depolarization, Ca^2+^ overload, and cell death [8–17]. Since mitochondrial health is recognized as a feature essential for optimal viability of biological systems, interventions designed to protect renal mitochondria during CS are worth investigating as potential therapeutic approaches. For example, our earlier studies revealed that overexpression of the mitochondrial antioxidant, manganese superoxide dismutase (MnSOD), in renal tubular cells confers protection from CS-induced renal injury [10]. Similarly, the addition of the mitochondrial-targeted antioxidant, mito-ubiquinone (MitoQ), to the CS solution is renoprotective *in vitro* and *ex vivo* [15–16]. These findings highlight a contribution of mitochondrial ROS and respiratory dysfunction to CS-induced renal injury however, identification of specific mitochondrial target proteins involved in CS induced renal injury is lacking.

Excitingly, we recently reported using an *in vitro* model of renal CS followed by rewarming (CS+RW) that pharmacological activation of the mitochondrial large (or Big) conductance Ca^2+-^activated K^+^ channel (mitoBK) during CS, by adding the specific BK activator NS11021 to the CS solution, protected rat kidney proximal tubular cells against CS+RW -induced mitochondrial injury (including respiratory dysfunction, superoxide production, and depolarization) and cell death in a mitoBK-dependent manner [18]. The mitoBK channel resides in the inner mitochondrial membrane (for review, see Balderas et al. [19]) and has been identified in many cell types using *in vitro* and *in vivo* models.

The purpose of the current study was to evaluate whether CS+Tx leads to a loss of mitoBK channels and whether pharmacological activation of the mitoBK channel during CS protects transplanted rat kidneys against CS+Tx-induced mitochondrial and renal injury. To carry out these *in vivo* preclinical studies, we used our well-established syngeneic rat model of renal CS+Tx [11–12, 20]. Prior to transplantation, the specific BK activator, NS11021, was added to the CS solution (3 μM) to duration of CS. We report for the first time that mitoBK channels are expressed in rat kidney mitochondria and its expression is reduced after CS+Tx. Excitingly, addition of the specific BK channel activator, NS11021, to the CS solution partially protects transplanted rat kidneys against CS+Tx-induced mitochondrial respiratory dysfunction, oxidative protein nitration, and cell death.

## 2. Materials and Methods

### 2.1. Ethical Approval

The animal use protocol (AUP #3837) was approved by the Institutional Animal Care and Use Committee (IACUC) at the University of Arkansas for Medical Sciences (UAMS), and all animal experiments were performed in compliance with institutional and National Institutes of Health guidelines.

### 2.2. Syngeneic Rat Model of Renal Cold Storage Followed by Transplantation (CS+Tx)

Orthotopic renal transplant surgery was performed in 3-month old male Lewis rats as recently described [11–12, 20]. Briefly, for the donor rat surgery, rats were anesthetized using isoflurane, and the kidneys were flushed with saline (+ 0.1% DMSO or 3 μM NS11021) and stored in University of Wisconsin (UW) CS solution at 4 °C for 18 hr. CS solution contained either Vehicle (0.1% DMSO; n= 3) or NS11021 (3 μM; n=3) depending on the intended treatment group. For the recipient rat surgery, male Lewis rats were anesthetized using isoflurane, the native left kidney was removed, and the donor left kidney (exposed to CS −/+ NS11021) was transplanted using end-to-end anastomosis. The surgical ischemia time was less than 45 min. The right native kidney was immediately removed, making renal function dependent on the transplanted left kidney. The ureter was anastomosed end-to-end over a 5 mm PE-50 polyethylene stent. Postoperatively, the animals were given 0.9% (w/v) NaCl in the abdominal cavity and placed under a heating lamp to recover from the anesthesia. All efforts were made to minimize suffering, and pain medication (buprenorphine 0.06 mg/kg, SC) and post-operative care were delivered as previously described and according to our AUP and IUCUC policy [11]. After 24 hr of reperfusion, the transplanted left kidney and blood were collected under anesthesia and saved as the cold storage plus transplantation (CS+Tx) group (n=3). Transplanted kidneys exposed to NS11021 during CS were saved as the CS+Tx + NS11021 group (n=3).

Rats used for Sham surgery underwent identical surgery (right nephrectomy), but without the renal transplantation. The right kidney from a healthy rat was removed. The left kidney remained functioning for 24 hr, and then the sham kidney and blood were harvested and saved as the Sham group (n=3). The ‘sham’ kidney served as the control for the CS+Tx model since all underwent a nephrectomy (removal of right kidney). After harvesting kidneys from 24 hr after sham or CS+Tx surgery, kidneys were immediately processed for HRR studies, mitochondrial isolation, or histology (formalin fixed).

### 2.3. Isolation of Rat Kidney Mitochondria

Mitochondrial fractions from freshly minced rat kidney tissues were prepared by douncehomogenization followed by differential centrifugation in a mannitol-sucrose buffer (pH 7.4) composed of (in mM): (mannitol 225, sucrose 75, HEPES 10, EGTA 0.1). Briefly, rat kidney homogenates were pelleted twice to remove nuclei, cell debris, and remaining intact cells (750 g, 10 min, 4°C). Contaminating lysosomes were separated from the homogenate supernatant by centrifugation at 3,000 g (15 min, 4°C). Subsequently, the cytosolic and mitochondrial fractions were separated by centrifugation at 7,000 g (15 min, 4°C). The cytosolic fraction (supernatant) was subjected to ultracentrifugation (100,000 g, 30 min, 4°C) and then flash-frozen in liquid nitrogen and stored at −80°C until use. In parallel, the mitochondrial fraction (7,000 g pellet described above) was subjected to two more spin-wash steps (7,000 g) and then flash-frozen in liquid nitrogen and stored at −80°C until use. All steps of the mitochondrial fractionation procedure were performed at 4°C.

### 2.4. Protein Lysates and Immunoblotting

Protein lysates from rat kidney mitochondrial fractions were prepared by using radioimmuno-precipitation assay (RIPA) buffer composed of (in mM, from Sigma, USA): phenylmethylsulfonyl fluoride (PMSF) 1, dithiothreitol (DTT) 1, and 1x Halt™ protease and phosphatase inhibitor cocktail (Thermoscientific, USA, #78442). Protein concentrations were determined by Coomassie Plus™ Protein Assay Reagent (Thermoscientific, USA, #23236). Mitochondrial fraction-derived protein lysates (20 μg) or cytosolic fraction protein extracts (20 μg) were resolved via SDS-PAGE (200V; 30 min) using precast Bolt™ 8% Bis-Tris Plus gels (Invitrogen, USA, #NW00080BOX) followed by wet-tank electrophoretic transfer (100V; 2 hr) to a PVDF membrane. Following transfer, membranes were blocked with 5% non-fat dry milk in TBS-T (0.05% Tween-20) for 1 hour at room temperature. Western blotting was performed using antibodies against the following proteins: the pore-forming subunit of the BK channel, BKα (1:1000; Alomone APC-107), NADH:Ubiquinone Oxidoreductase Core Subunit S3 of Complex I, NDUFS3 (1:1000; Abcam ab110246), proteasome subunit beta type-5, PSMB5 (1:1000; Abcam ab3330), and β-actin (1:1000; Sigma A5441). NDUFS3 expression was used as a mitochondrial loading control and marker and PSMB5 expression was used as a cytosolic marker. β-Actin expression was used as a standard loading control. The probed membranes were washed three times and immunoreactive proteins were detected using horseradish peroxidase -conjugated secondary antibodies (1:30,000; Seracare KPL, USA) and enhanced chemiluminescence (SuperSignal™ West Pico PLUS Chemiluminescent Substrate, Thermoscientific, USA, #34580). Densitometry was calculated using AlphaEase FC software.

### 2.5. High Resolution Respirometry (HRR)

Mitochondrial respiratory complex activity was measured in saponin-permeabilized rat kidney biopsies by high resolution respirometry (HRR) (Oroboros instruments—Oxygraph-2k, Innsbruck, Austria) using a substrate-inhibitor-titration (SIT) protocol as described by our previous reports [11–12]. The SIT protocol enables measurement of oxidative phosphorylation-dependent O2 flux (mitochondrial respiration) for each individual complex (Complexes I–IV) of the electron transport chain (ETC).

Briefly, representative renal biopsies (5–7 mg tissue containing cortex and medulla) were excised from rat kidneys 24 hours after sham surgery or CS+Tx surgery, briefly washed once with MiRO5 buffer (pH, 7.0) composed of (in mM): K-lactobionate 60, EDTA 0.5, MgCl2 3.0, taurine 20, KH2PO4 10, HEPES 20, sucrose 110, and 1 g/L BSA), then permeabilized with 100 μg/mL saponin in MiRO5 buffer for 30 minutes at 4°C, and subsequently washed three more times in MiRO5 buffer. The permeabilized renal biopsies were then added to the Oxygraph chamber containing O2-saturated MiRO5 medium at 37°C.

Mitochondrial respiration was initiated by adding 2 mM malate and 10 mM glutamate, and maximal oxidative phosphorylation-dependent respiration was achieved by adding 2.5 mM ADP. Subsequently, rotenone (100 μM) was added to completely inhibit complex I respiration. Complex II and III respiration was initiated by adding 10 mM succinate followed by 2 mM malonate to inhibit Complex II respiration and subsequently 10 μM antimycin A to inhibit complex III respiration. Complex IV respiration was stimulated by adding 5 μM Tetramethyl-*p-*phenylenediamine (TMPD; ascorbate-stabilized) followed by inhibition with 250 mM azide. Inhibitor concentrations were selected based on previous experimental determination of the concentrations required to maximally reduce substrate-induced respiration [11–12]. The DATLAB 4.2 software (Oroboros) was used for data analysis, and mitochondrial respiration for each individual ETC complex was expressed as oxygen flux (pmol/mg tissue/s).

### 2.6. Evaluating Cell Death with Transferase-mediated dUTP Nick-end Labeling (TUNEL) Assay

Cell death was visualized using the in situ terminal transferase-mediated dUTP nick-end labeling (TUNEL) method and was utilized according to the protocol provided by the manufacturer (TACS TdT Kit, R&D Systems, MN). Counterstaining was performed using Gill’s hematoxylin. Cell death was semi-quantitatively evaluated by the abundance of TUNEL-positive nuclei in randomized fields for blind-labeled tissue sections as shown previously [16, 21]. Briefly, the number of TUNEL-positive nuclei per field (400x) was counted in 7 randomized fields for each tissue section. Each animal’s overall score was averaged from those randomized fields.

### 2.7. Protein Tyrosine Nitration Immunohistochemistry

Oxidative stress was measured using an anti-nitrotyrosine antibody and immunohistochemical analysis as previously outlined by our laboratory [15–16]. Briefly, paraffin-embedded kidney tissue sections were deparaffinized in xylenes, xylenes were progressively washed in 100%, 95%, and 70% ethanol, and hydrated in ddH2O. Antigens were retrieved by heating sections in 10 mM sodium citrate buffer (pH 6.0) in a sub-boiling hot water bath for 20 min at 98 °C. Endogenous peroxidase was quenched by incubating the sections with Peroxidase Suppressor (Thermo Scientific, Rockford, IL, USA) for 15 min at RT. The slides were blocked with Non Serum Protein Block (Dako, Carpinteria, CA, USA) for 20 min at RT. Primary antibodies were prepared in antibody diluent solution (0.5% nonfat dry milk and 1% BSA in TBS) and incubated overnight at 4 °C. The primary antibody Anti-Nitrotyrosine was used at a 1:1000 dilution (Millipore, Temecula, CA, USA). The specificity of nitrotyrosine antibody binding in the renal tissue was confirmed by blocking the antibody with 3-nitrotyrosine (10 mM). Immunoreactivity was detected by Dako Envision+ System-HRP (Dako, Carpinteria, CA, USA). Semi-quantitative scoring of nitrotyrosine staining intensity (on a scale from 1–5, with 1 being the lowest and 5 being highest nitrotyrosine intensity) was performed in a blinded manner using 10 randomized fields (400x) of tissue sections, as shown previously [16, 21–23]. Each animal’s overall score was averaged from those randomized fields.

### 2.8. Measuring Renal Function via Blood Chemistry

Blood chemistry was determined in heparinized blood (arterial) using a hand-held clinical chemistry analyzer, iSTAT, and cartridges (CHEM8+) as described by the manufacturer (Vetscan®, Abaxis, USA).

### 2.9. Statistical Analysis

Results are presented as mean ± standard error of the mean (SEM) (GraphPad Prism 8 software). Data were analyzed by one-way ANOVA and Tukey’s post-hoc test for multiple group comparisons. Differences at the *P* < 0.05 level were considered statistically significant.

## 3. Results

### 3.1. MitoBK Channels are Expressed in Rat Kidney Mitochondrial Fractions and its Protein Level is Reduced after CS+Tx

**Figure 1.**
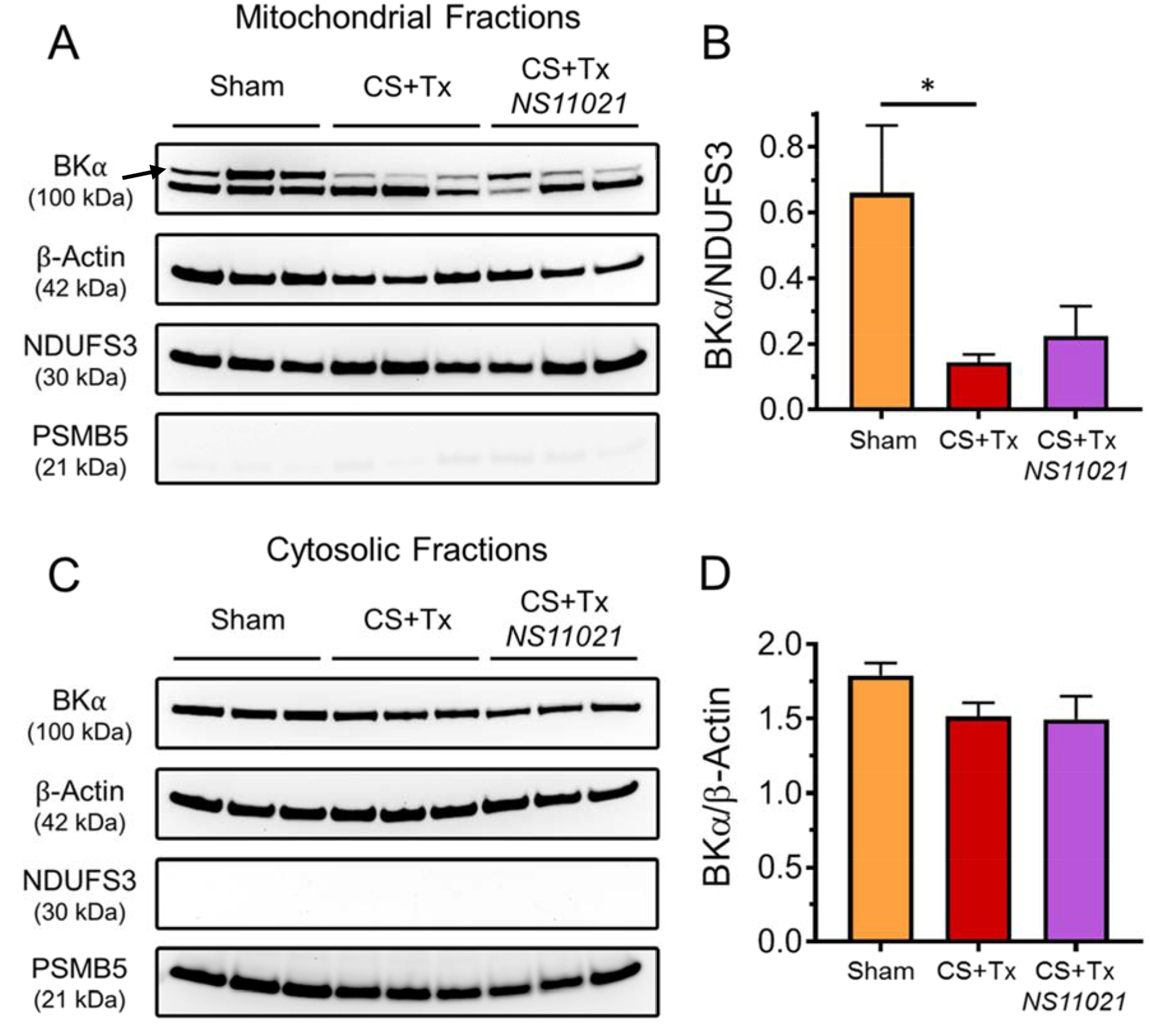
CS+Tx diminishes BK*α* protein level in rat kidney mitochondrial fractions. Western blot shows protein expression of the pore-forming BK*α* subunit (top band indicated by black arrow) in mitochondrial fractions (A) and cytosolic fractions (C) from rat kidneys after sham surgery or CS+Tx. β-Actin and NDUFS3 served as cytosolic and mitochondrial loading controls, respectively. Representative blot shown where each lane is loaded with 25 μg protein corresponding to a separate experiment. Densitometry of BKα protein level for the mitochondrial fractions (B) and cytosolic fractions (D) are shown next to corresponding blot; values are expressed as mean ± SEM (n=3), *P<0.05.

We used Western blotting to verify the presence of the BK channel in mitochondria of sham rat kidneys and to determine whether the protein level of the mitoBK channel is altered after cold storage followed by transplantation (CS+Tx). The protein expression of the pore-forming α subunit of the BK channel (BKα) was detected in rat kidney mitochondrial fraction protein lysates as a doublet of 100 kDa and 90 kDa bands (Figure 1A). Our recent study using rat renal cells and other reports have focused on the higher MW mitoBKα band observed (100 kDa, indicated by arrow) [18–19, 24]. The lower MW 90 kDa mitoBKα band is of unknown identity and has not been observed in isolated mitochondria to our knowledge, though it may be a splice variant or a cleaved, incomplete form of mitoBKα. BKα expression (100 kDa) was also detected in rat kidney cytosolic fractions with no evident change after CS+Tx (Figure 1C). NSUFS3, a subunit for NADH dehydrogenase (Complex I), was used as the mitochondrial marker and loading control, PSMB5 (20S proteasome subunit beta-5) was used as a cytosolic marker, and β-actin was used as a loading control for cytosolic fractions. Selective expression of NDUFS3 in the mitochondrial fractions and of PSMB5 in the cytosolic fractions confirmed the correct isolation of mitochondrial/cytosolic subcellular fractions of rat kidney tissue.

Densitometry showed BKα protein level (top band, 100 kDa) is severely reduced after CS+Tx (~80% reduction) in rat kidney mitochondrial fractions (Figure 1B). Protein level of the 90 kDa BKα band in mitochondrial fractions was unchanged after CS+Tx (data not shown). The addition of the selective BK activator, NS11021 (3 μM), to the CS solution had no impact on mitoBKα protein level in transplanted rat kidneys (CS+Tx + NS) compared to those without NS11021 treatment (CS+Tx). Protein level of BKα, however, was unaffected by CS+Tx in rat kidney cytosolic fractions (Figure 1D). The presence of the BKα subunits detected in rat kidney cytosolic fractions may be attributed to persisting membrane fragments in the cytosol originating from non-mitochondrial organelles and the plasma membrane. Overall, these data provide novel evidence that the mitoBK channel is expressed in rat kidney and that its protein level appears to be profoundly diminished after CS+Tx.

### 3.2. NS11021 Mitigates CS+Tx -induced Mitochondrial Respiratory Dysfunction at Complexes II-IV

**Figure 2.**
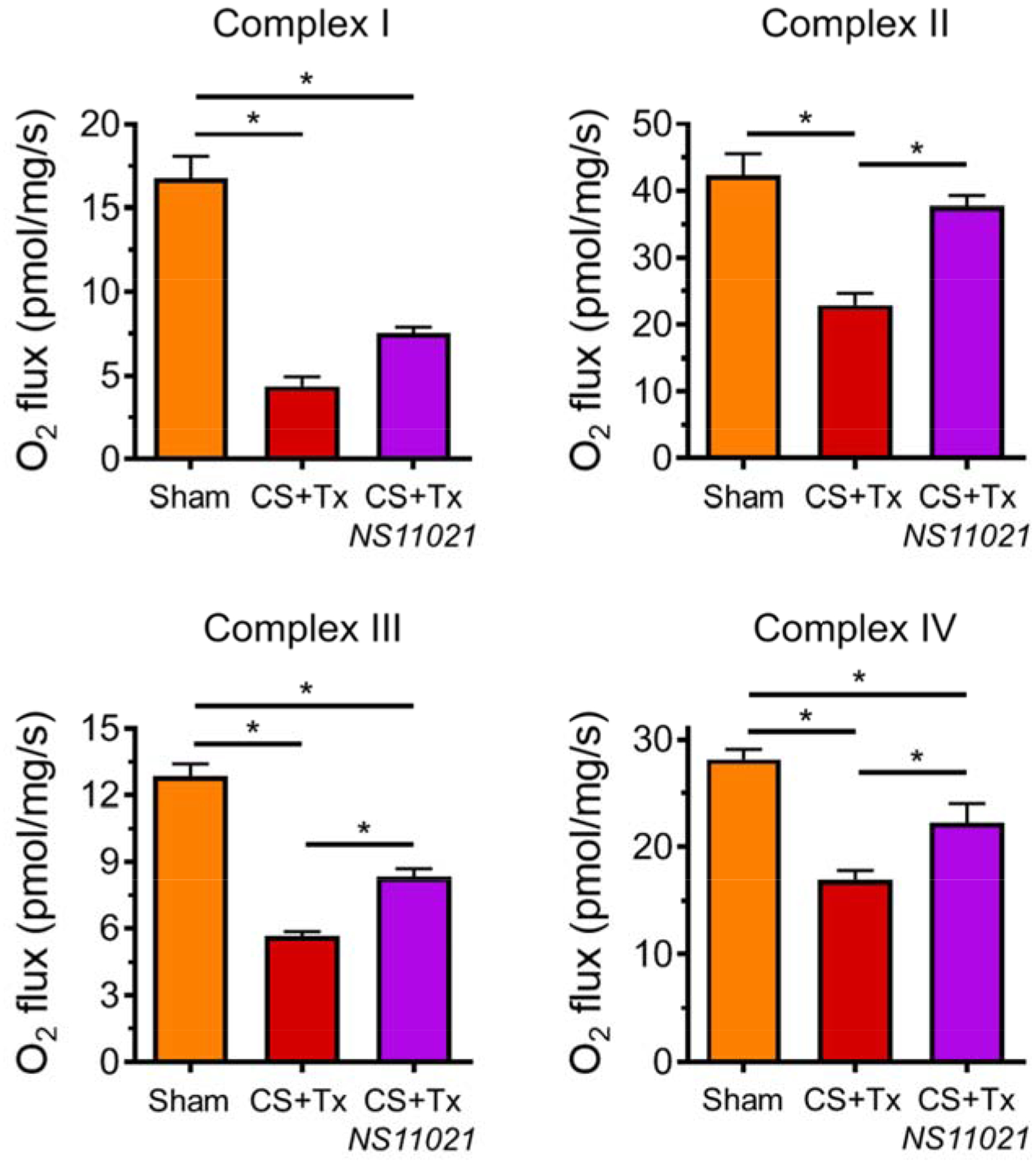
NS11021 protects against CS+Tx-induced mitochondrial respiratory dysfunction at Complexes II-IV in rat kidney. HRR was used to measure mitochondrial respiration (O_2_ flux) at Electron Transport Chain Complexes I-IV in rat kidney tissue biopsies (normalized per mg tissue). Data is shown for the following conditions: sham, after CS+Tx, and after CS+Tx treated with NS11021 (3 μM) during CS; values are expressed as mean ± SEM (n=3), *p<0.05.

High resolution respirometry (HRR) was used to determine whether treatment with the BK activator, NS11021, during CS protects against CS+Tx-induced rat kidney mitochondrial respiratory dysfunction. Compared to Sham, CS+Tx impaired renal mitochondrial respiration at Complexes I-IV by 70%, 40%, 55%, and 40%, respectively (Figure 2). CS+Tx-induced renal mitochondrial respiratory dysfunction at complexes I-IV in the current study is consistent with our recent findings [11]. Addition of NS11021 (3 μM) during CS (18 hr) followed by Tx (24 hr reperfusion) was fully protective against CS+Tx-induced respiratory dysfunction at Complex II and was partially protective at Complexes III and IV.

### 3.3. NS11021 Protects Against CS+Tx –induced Protein Tyrosine Nitration

**Figure 3.**
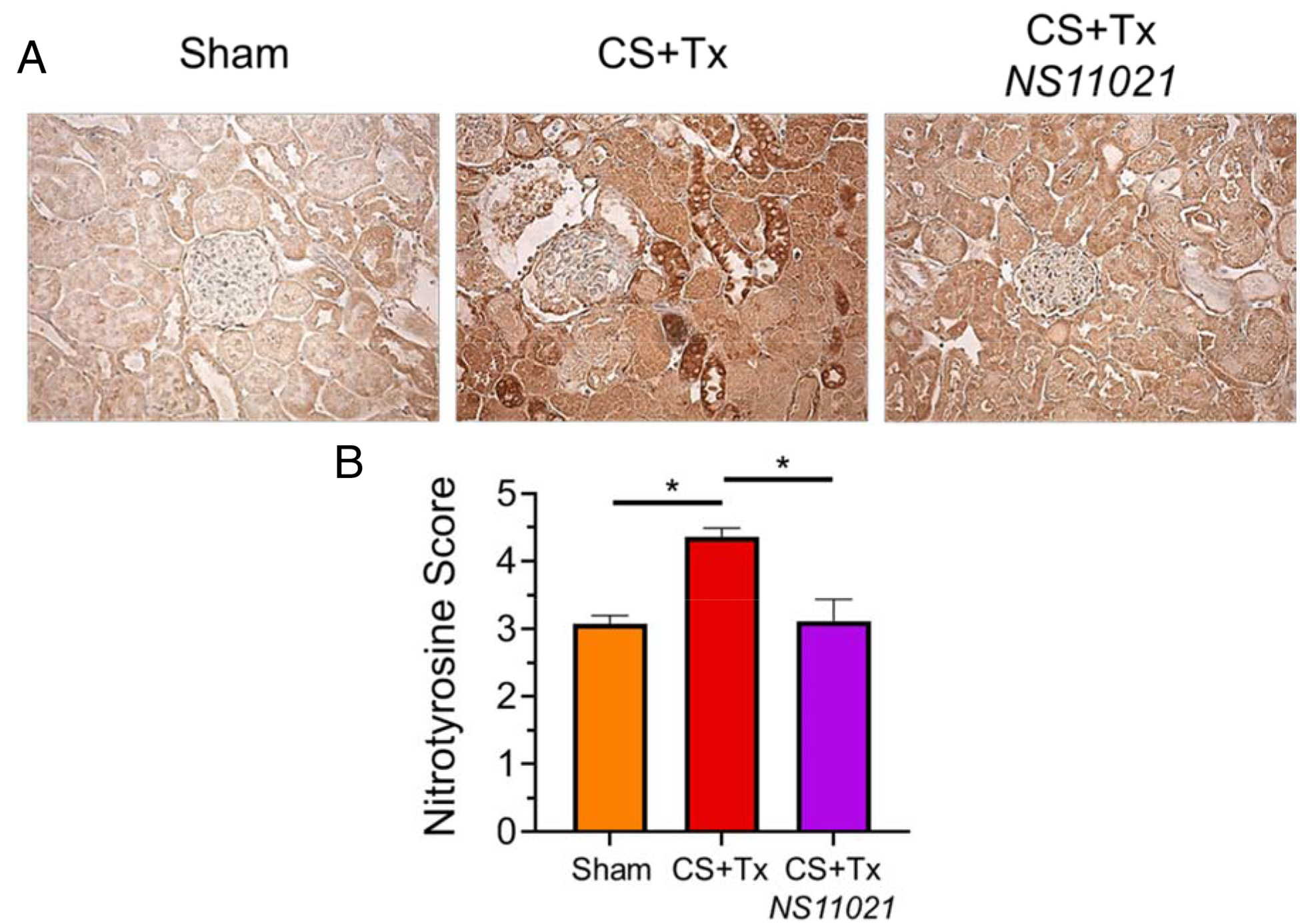
NS11021 protects against CS+Tx-induced protein tyrosine nitration. (A) Brightfield microscopy 200x images of representative renal tissue sections show anti-nitrotyrosine immunohistochemical staining. Data is shown for the following conditions: sham, after CS+Tx, or after CS+Tx treated with NS11021 (3 μM). (B) Ten Randomized fields of blind-labeled renal tissue sections were semi-quantitatively scored for nitrotyrosine intensity level using a scale of 1–5; values are expressed as mean ± SEM (n=3), *P<0.05.

Protein tyrosine nitration is a common indicator of oxidative injury [25–26] and was assessed via immunohistochemistry in rat kidney tissue sections using an anti-nitrotyrosine antibody. Nitrotyrosine intensity was semi-quantitatively scored in a blinded manner using 10 randomized fields for each tissue section. Brightfield microscopy (200x) pictures of representative cortical kidney tissue sections show nitrotyrosine immunohistochemical staining for sham rats and CS+Tx −/+ NS11021 rats (Figure 3A). Sham kidneys displayed modest levels of nitrotyrosine as expected due to the stress of sham (nephrectomy) surgery [21]. Nitrotyrosine staining intensity increased following CS+Tx when compared to the Sham rat kidneys (Figure 3B). Rat kidneys treated with NS11021 during CS show markedly reduced nitrotyrosine staining after transplantation compared to untreated CS+Tx kidneys and were not significantly different than sham kidneys. Clearly, addition of NS11021 to the CS solution was protective against CS+Tx-induced protein tyrosine nitration.

### 3.4. NS11021 Attenuates CS+Tx –induced Cell Death

TUNEL staining, which detects DNA fragmentation generated during cell death such as apoptosis and necrosis, was used in rat kidney tissue sections to determine whether NS11021 treatment during CS protects against CS+Tx-induced cell death. In these experiments, TUNEL-positive nuclei, which indicate cell death, were semi-quantitatively scored in a blinded manner using 7 randomized fields for each tissue section.

**Figure 4.**
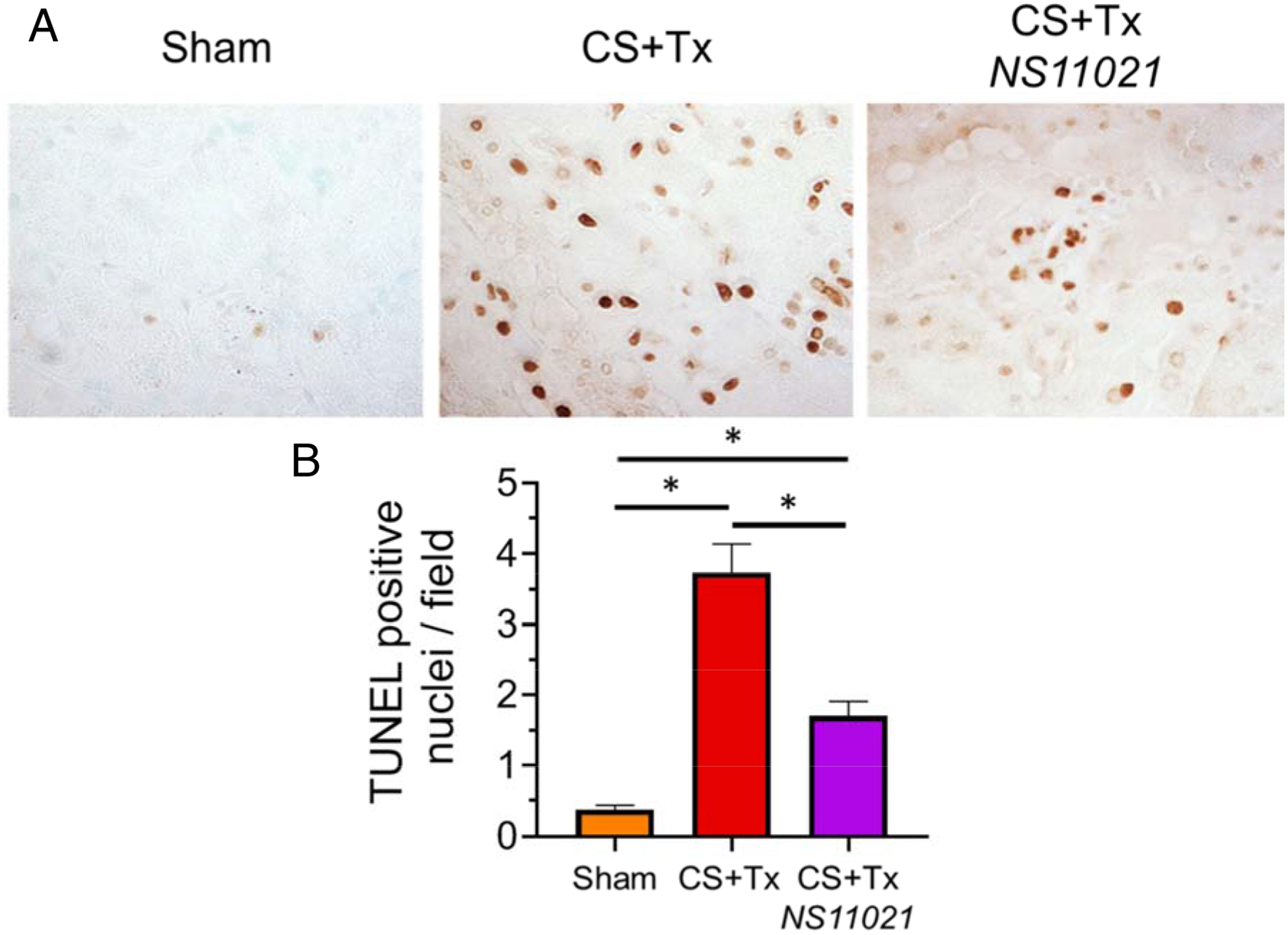
NS11021 protects against CS+Tx-induced cell death as indicated by TUNEL-positive nuclei. (A) Brightfield microscopy 200x images of representative renal tissue sections show histological TUNEL staining. Data is shown for the following conditions: sham, after CS+Tx, or after CS+Tx treated with NS11021 (3 μM). (B) Randomized fields of blind-labeled renal tissue sections (7 fields) were semi-quantitatively scored for number of TUNEL-positive nuclei/field; values are expressed as mean ± SEM (n=3), *P<0.05.

Brightfield microscopy (200x) pictures of representative renal tissue sections show TUNEL staining for sham rats and CS+Tx −/+ NS11021 rats (Figure 4A). The number of TUNEL-positive nuclei per field is minimal in sham kidneys and was significantly elevated 10-fold after CS+Tx (0.37 vs 3.7, respectively) (Figure 4B). NS11021 treatment during CS significantly lowers CS+Tx-induced TUNEL-positive nuclei per field by about 55% (1.7 vs 3.7, respectively). The TUNEL assay data indicate that NS11021 treatment during CS partially protects against CS+Tx-induced cell death.

### 3.10. NS11021 Does Not Protect Against CS+Tx –induced Acute Renal Dysfunction as Indicated by SCr and BUN

**Figure 5.**
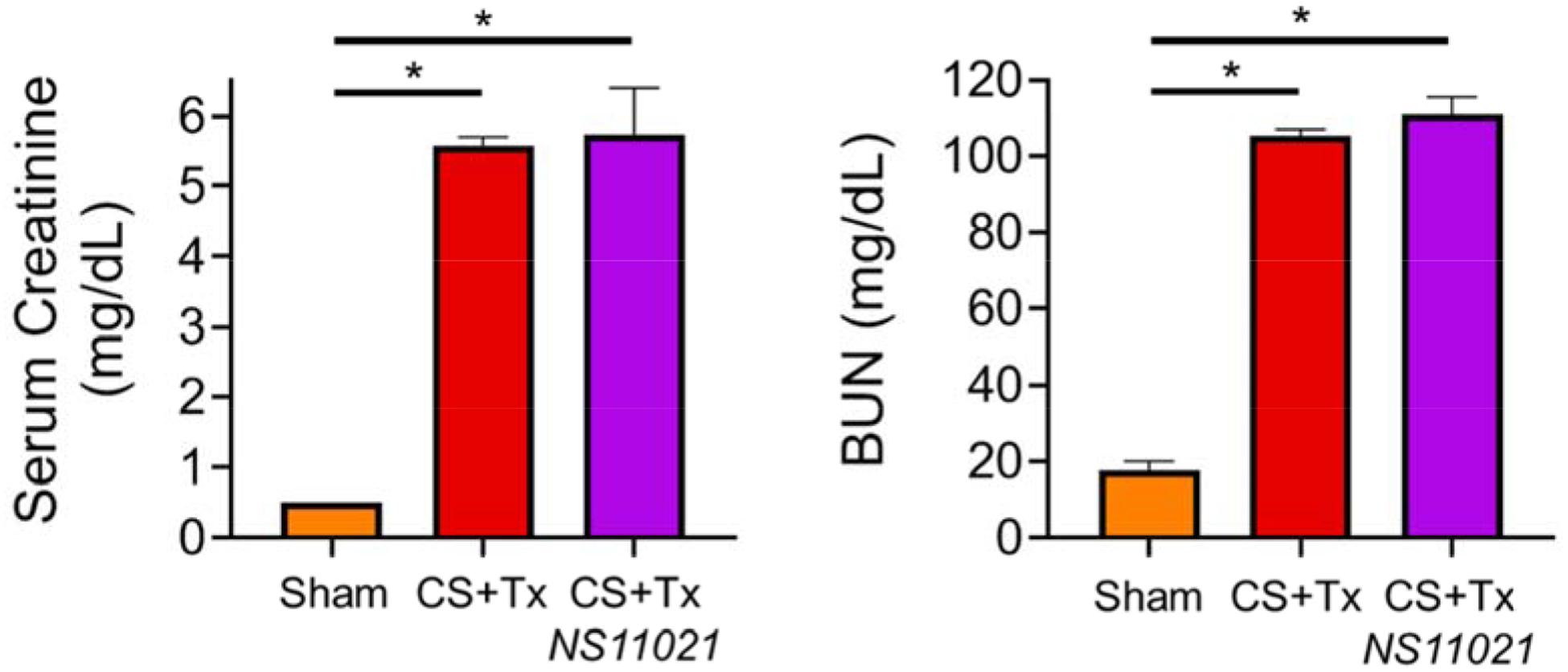
NS11021 does not prevent CS+Tx-induced rat renal dysfunction. Serum Creatinine and Blood Urea Nitrogen (BUN) levels are shown for rats after sham surgery or CS+Tx −/+ NS11021 treatment; values are expressed as mean ± SEM (n=3), *P<0.05 vs sham.

Serum Creatinine (SCr) and Blood Urea Nitrogen (BUN), which are widely used biomarkers for renal dysfunction, were measured in rats after sham surgery or after CS+Tx to determine whether NS11021 treatment protected against CS+Tx-induced renal dysfunction. SCr and BUN levels were measured using iSTaT cartridges as described in methods and our previous report [11]. Compared to sham surgery, CS+Tx severely elevated levels of SCr (0.5 vs 5.5 mg/dL) and BUN (20 vs 105 mg/dL), which is consistent with our previous reports (Figure 5) [11]. However, NS11021 treatment during CS was not protective against CS+Tx-induced acute renal dysfunction as indicated by SCr and BUN at an early 24 hr reperfusion time point.

## 4. Discussion

The present study has three primary findings. We show, for the first time in an *in vivo* model, that mitoBK channels are expressed in rat kidney mitochondria. We also provide evidence indicating that renal mitoBK channel protein expression is lowered after cold storage followed by transplantation (CS+Tx), and the loss of mitoBK channels after CS+Tx suggests it may be a relevant therapeutic target. Third, and most importantly, our findings indicate that addition of the BK channel opener, NS11021, to the CS solution partially protects rat kidneys against key parameters of CS+Tx -induced renal injury, including mitochondrial respiratory dysfunction, protein tyrosine nitration, and cell death, but not acute renal dysfunction (as indicated by SCr and BUN). Collectively, this preclinical study raises the possibility that new strategies involving addition of pharmacological BK channel activators, such as NS11021, to the CS solution may protect against CS+Tx-induced mitochondrial and renal injury.

### 4.1. MitoBK Channels are Expressed in Rat Kidney and its Protein Level is Diminished After CS+Tx

This is the first study to verify protein expression of the mitoBK channel in rat kidney. Rat kidney tissue mitochondrial fractions expressed a 100-kDa immunoreactive band on Western blots corresponding to the pore-forming BKα subunit. Interestingly, protein levels of mitoBKα was reduced after CS+Tx. Considering the endogenously-protective role that mitoBK channels have during IR-related injuries [19, 27–28], we propose the loss of mitoBK channels contributes to CS+Tx-induced mitochondrial and renal injury. Accordingly, we anticipated that pharmacologically targeting the mitoBK channel to enhance its activity during CS with the use of NS11021 would be similarly protective *in vivo* as we recently reported using our renal cell CS+RW model [18].

While our *in vitro* and *in vivo* models similarly express mitoBK channels, a notable difference is that CS+Tx greatly reduced mitoBK protein level *in vivo* whereas CS+RW had no impact on mitoBK protein level *in vitro* [18]. A possible explanation for this is that our rat CS+Tx renal injury is clearly a more severe model due to injurious contributions from surgical trauma, vascular damage, and a systemic inflammatory response.

### 4.2. NS11021 Partially Protects Against CS+Tx-induced Mitochondrial and Renal Cell Injury

Excitingly, we observed that addition of NS11021 to the CS solution protected transplanted donor kidneys from CS+Tx -induced injury, including mitochondrial respiratory dysfunction, protein tyrosine nitration, and cell death. These subcellular injury parameters are critical therapeutic endpoints to evaluate since they pathophysiologically contribute to CS+Tx -induced renal dysfunction and ultimately renal failure [8–17]. Overall, these *in vivo* preclinical findings are consistent with our previous report where addition of NS11021 to the CS solution protected against CS-induced –induced mitochondrial injury and cell death in an *in vitro* model of renal CS [18].

Disappointingly, NS11021 did not protect against CS+Tx-induced acute renal dysfunction as indicated by the sustained elevation of SCr and BUN. However, we would like to highlight that (1) the renal dysfunction observed with our rat CS+Tx model is quite profound (SCr > 5), (2) our CS+Tx rat model utilized a short reperfusion phase of only 24 hours, and (3) SCr and BUN are indirect markers for detecting recovery of renal function after acute injury [29–30]. We speculate that a longer reperfusion phase of 3–7 days may be necessary to observe any potential recovery of renal function (as indicated by SCr and BUN) following transplantation with kidneys treated with NS11021 during CS. These explanations are plausible considering that renal function on the organ-level ultimately depends on a properly functioning multifaceted set of factors from the subcellular to tissue level, such as mitochondrial function, oxidative stress, cell death, tubular function, and countless more. Thus, recovery of renal function may lag behind these (sub)cellular parameters where we observed protection with NS11021 treatment.

The conceptual rationale behind our therapeutic approach is to pharmacologically activate mitoBK channels in donor kidneys during the CS period, which will protect against CS+Tx -induced mitochondrial and renal injury. A key advantage of this therapeutic intervention is that *only* the donor kidney is exposed to the drug during CS. Thus, the transplant recipient avoids systemic drug exposure and any potential systemic side effects which may otherwise complicate clinical implementation of promising therapies to mitigate CS renal injury.

### 4.3. Limitations and Pharmacological Considerations

The current study has several limitations that should be acknowledged. First, we only reported one concentration of NS11021 used in the CS solution (3 μM). We chose this concentration based on our recently published renal cell model that showed 1 μM NS11021 during CS was optimally protective [18]. We anticipated that a moderately higher concentration (3 μM) would be required to achieve therapeutic efficacy in the rat kidney compared to our recent *in vitro* study using a cell model of renal CS. In addition, while 3 μM NS11021 might seem high, we considered that adding NS11021 (which is hydrophobic) to the hyperosmotic CS solution at 4 °C may result in limited solubility and tissue diffusion compared to normothermic solutions, thus necessitating slightly higher concentrations. Nevertheless, prior studies have used up to 3 μM NS11021 for *ex vivo* models of cardiac IR injury and have yielded cardioprotection without adverse effects [31].

It should also be noted that the pharmacological effects of NS11021 may not be restricted to the mitoBK channel isoform considering that the plasmalemmal BK channel isoform is expressed in numerous cell types within the kidney. However, the current study infers that the pharmacotherapeutic effects of NS11021 are mediated by mitoBK, at least in part. This rationale is based on our recently published manuscript using a renal proximal tubular cell model (which expresses mitoBK but *not* the plasmalemmal BK isoform) where we found NS11021 to mitigate CS+RW-induced mitochondrial injury and cell death in a mitoBK-mediated manner [18]. That cell line was particularly useful for specifically evaluating mitoBK as a pharmacotherapeutic target without a potentially confounding plasmalemmal BK channel isoform. Thus, we anticipated that any mitoBK-mediated mitoprotective/cytoprotective effects with NS11021 would also extend *in vivo* with our CS+Tx model. However, we cannot discount the potential involvement of plasmalemmal BK-mediated effects with NS11021 treatment.

With the considerable loss of mitoBK protein expression after CS+Tx, it also seems likely that rat kidneys would suffer a loss of mitoBK function which may further contribute to CS+Tx injury. However, we were unable to assess mitoBK function in this study using the potassium-sensitive dye (PBFI) method as described in our recent paper using the renal cell CS+RW model [18] since these animal studies were performed before this assay was set up in the laboratory and the assay requires freshly isolated mitochondria. However, the purpose of this study was not to evaluate renal mitoBK function in detail, but rather to simply assess whether addition of NS11021 to the CS solution was protective against CS+Tx injury using a rat kidney transplant model.

Overall, these studies highlight the pharmacotherapeutic potential of targeting the mitoBK channel during CS to protect against CS+Tx-induced mitochondrial and renal cell injury. Accordingly, future preclinical studies that utilize longer time points, as well as deceased donor kidney models are warranted to optimize the pharmacological mitoBK-targeting process during CS for improved therapeutic efficacy, especially in regards to renal transplant function. If the improvement of long-term renal function can be demonstrated with this approach, then such a therapeutic intervention may be particularly clinically valuable due to its simplicity and lack of systemic drug exposure for patients.

## Supplementary Material

1

## Highlights:

- Rat kidney mitochondria express the mitoBK channel, a novel therapeutic target
- Clinically relevant rat renal cold storage (CS) and transplantation model was used
- Treatment included adding mitoBK activator NS11021 to kidney in CS solution
- NS11021 mitigates CS-induced mitochondrial injury after renal transplantation
- NS11021 attenuates oxidative stress and cell death after renal transplantation

## Abbreviations:

CS: cold storage
CS+Tx: cold storage followed by transplantation
mitoBK channel: mitochondrial large conductance Ca^2+^-activated K^+^ channel
CS+RW: cold storage followed by rewarming
NRK cells: normal rat kidney proximal tubular cells
HRR: High Resolution Respirometry
