# Supplementary material for: The BK Activator NS11021 Partially Protects Rat Kidneys from Cold Storage and Transplantation -Induced Mitochondrial and Renal Injury
Source: Arch Biochem Biophys. Author manuscript; Available in PMC 2021 Jul 30. (PMC7322695; doi:10.1016/j.abb.2020.108410)
Supplement: 1 [file NIHMS1601607-supplement-1.pdf]

The next slides show full western blot images (uncropped) that were used in **Figure 1**

- the membrane was cut at the 50 kDa marker for simultaneous probing of BKα and lower MW proteins
- Stripped and reprobed the lower MW blot (with <50 kDa proteins) as indicated

Figure 1
as it appears in
the manuscript

NDUFS3 – mitochondrial marker
& loading control

PSMB5 – cytosolic marker

β-Actin – cytosolic loading control

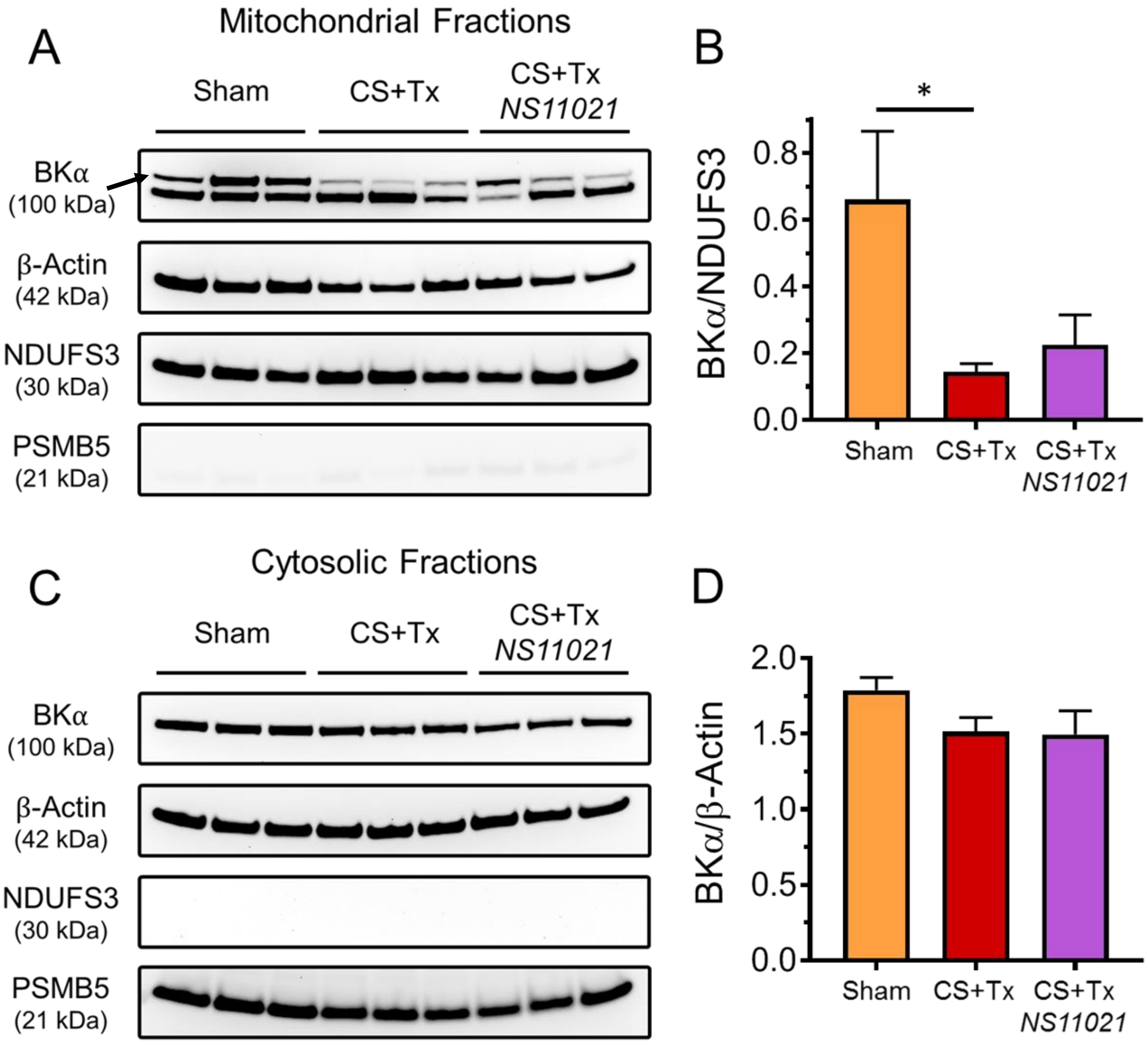

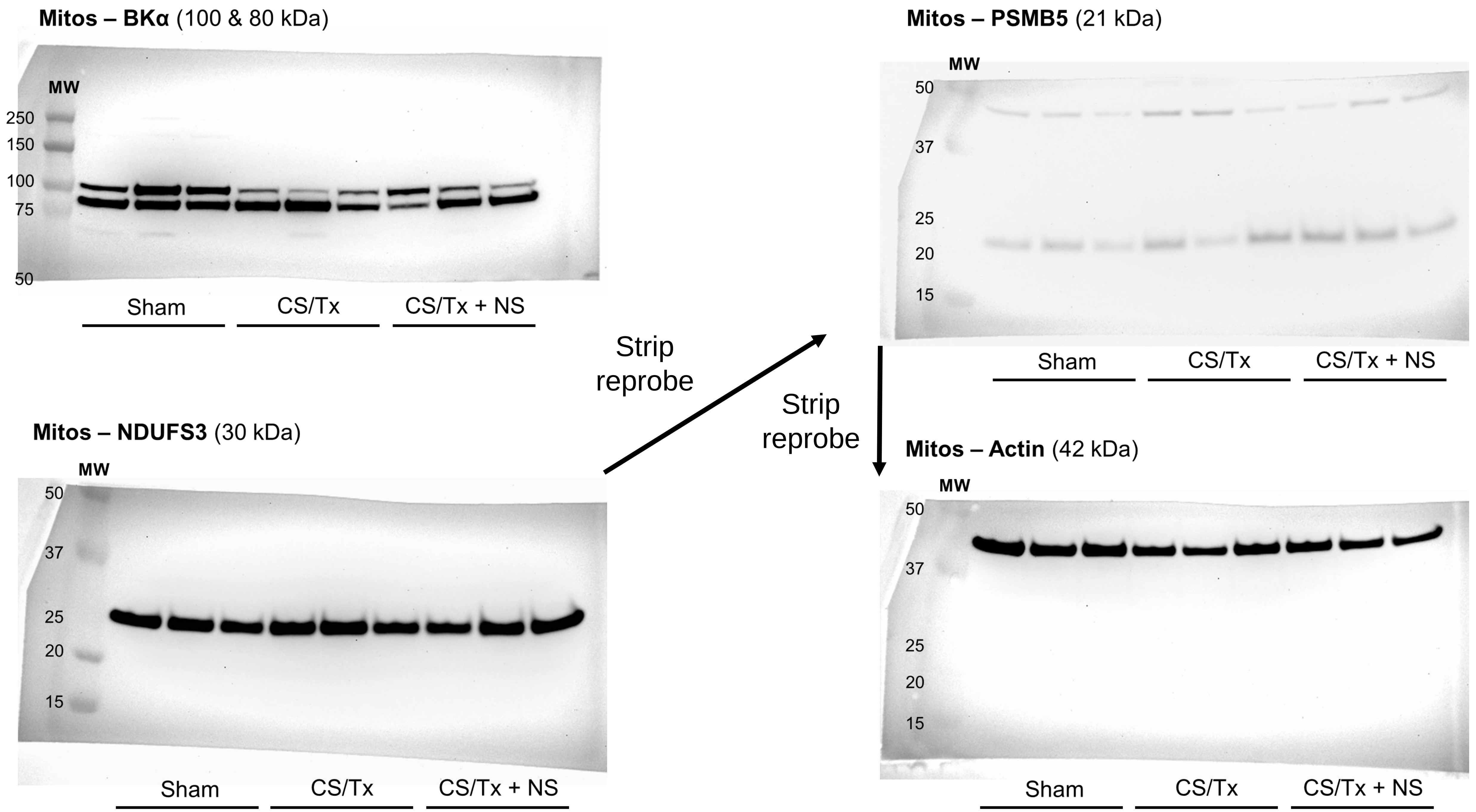

Mitos – BKα (100 & 80 kDa)
MW
250
150
100
75
50
Sham
CS/Tx
CS/Tx + NS
Strip
reprobe
Mitos – PSMB5 (21 kDa)
MW
50
37
25
20
15
Sham
CS/Tx
CS/Tx + NS
Strip
reprobe
Mitos – NDUFS3 (30 kDa)
MW
50
37
25
20
15
Sham
CS/Tx
CS/Tx + NS
Mitos – Actin (42 kDa)
MW
50
37
25
20
15
Sham
CS/Tx
CS/Tx + NS

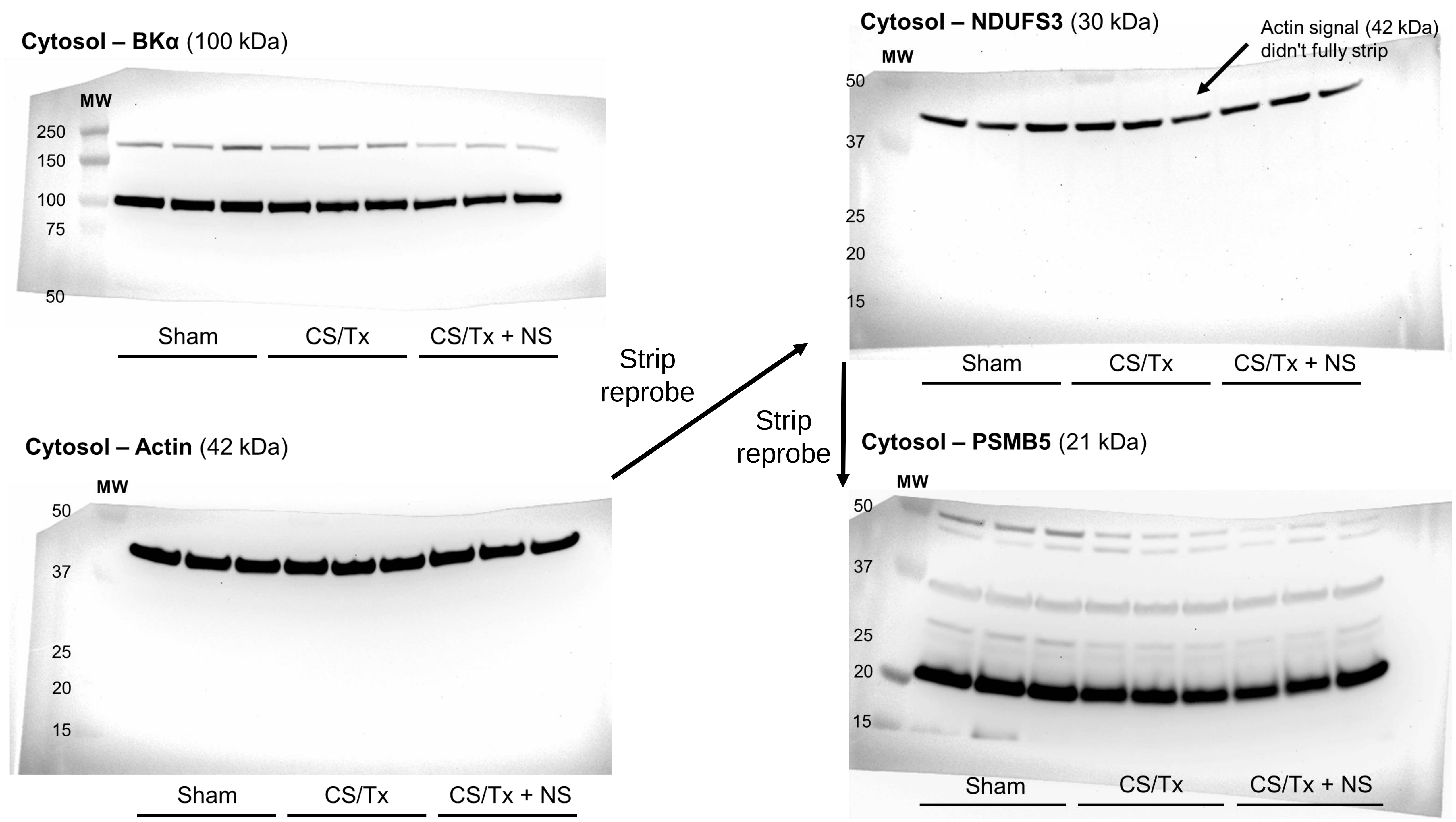

Cytosol – BKα (100 kDa)
MW
250
150
100
75
50
Sham
CS/Tx
CS/Tx + NS
Strip reprobe
Cytosol – NDUFS3 (30 kDa)
Actin signal (42 kDa) didn't fully strip
MW
50
37
25
20
15
Sham
CS/Tx
CS/Tx + NS
Cytosol – Actin (42 kDa)
MW
50
37
25
20
15
Sham
CS/Tx
CS/Tx + NS
Strip reprobe
Cytosol – PSMB5 (21 kDa)
MW
50
37
25
20
15
Sham
CS/Tx
CS/Tx + NS

HRR data

- raw numbers used in **Figure 2** y-axis (n=3)
- complex-specific O_2 flux (pmol / mg tissue / sec)

Complex I respiration		
**Sham**	**CS/Tx**	**CS/Tx + NS**
16.34	5.03	7.17
19.29	3.24	7.23
14.75	4.85	8.20

Complex II respiration		
**Sham**	**CS/Tx**	**CS/Tx + NS**
35.90	24.92	37.98
46.83	19.44	34.84
44.06	24.42	40.29

Complex III respiration		
**Sham**	**CS/Tx**	**CS/Tx + NS**
12.09	5.91	7.59
13.96	5.81	8.64
12.48	5.20	8.75

Complex IV respiration		
**Sham**	**CS/Tx**	**CS/Tx + NS**
27.24	18.58	23.21
30.02	16.63	19.02
27.18	15.53	24.69

Figure 2
as it appears in the manuscript

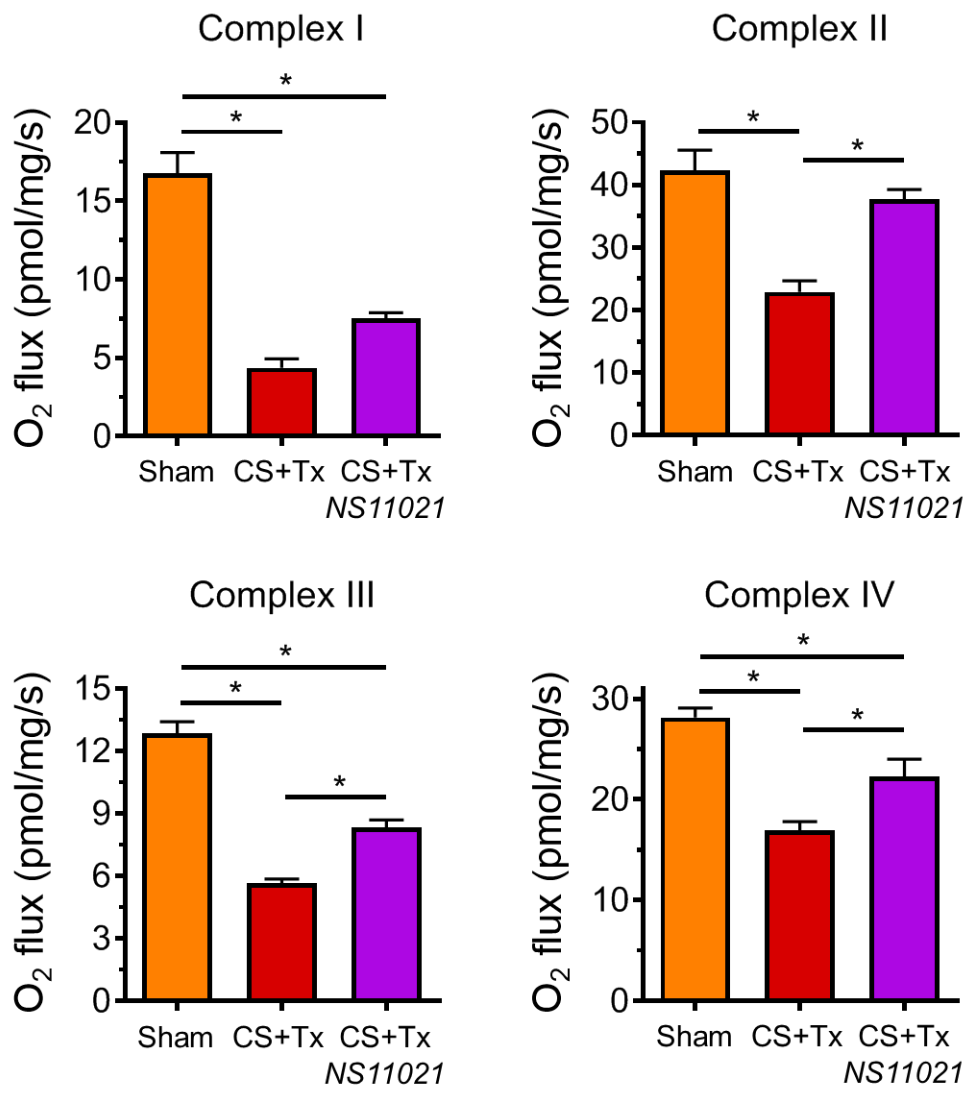

Example of HRR trace for a sham kidney
showing substrate-inhibitor titration protocol

Blue trace = oxygen concentration (nmol/mL)
Red trace = O2 flux (pmol / mg tissue / sec)

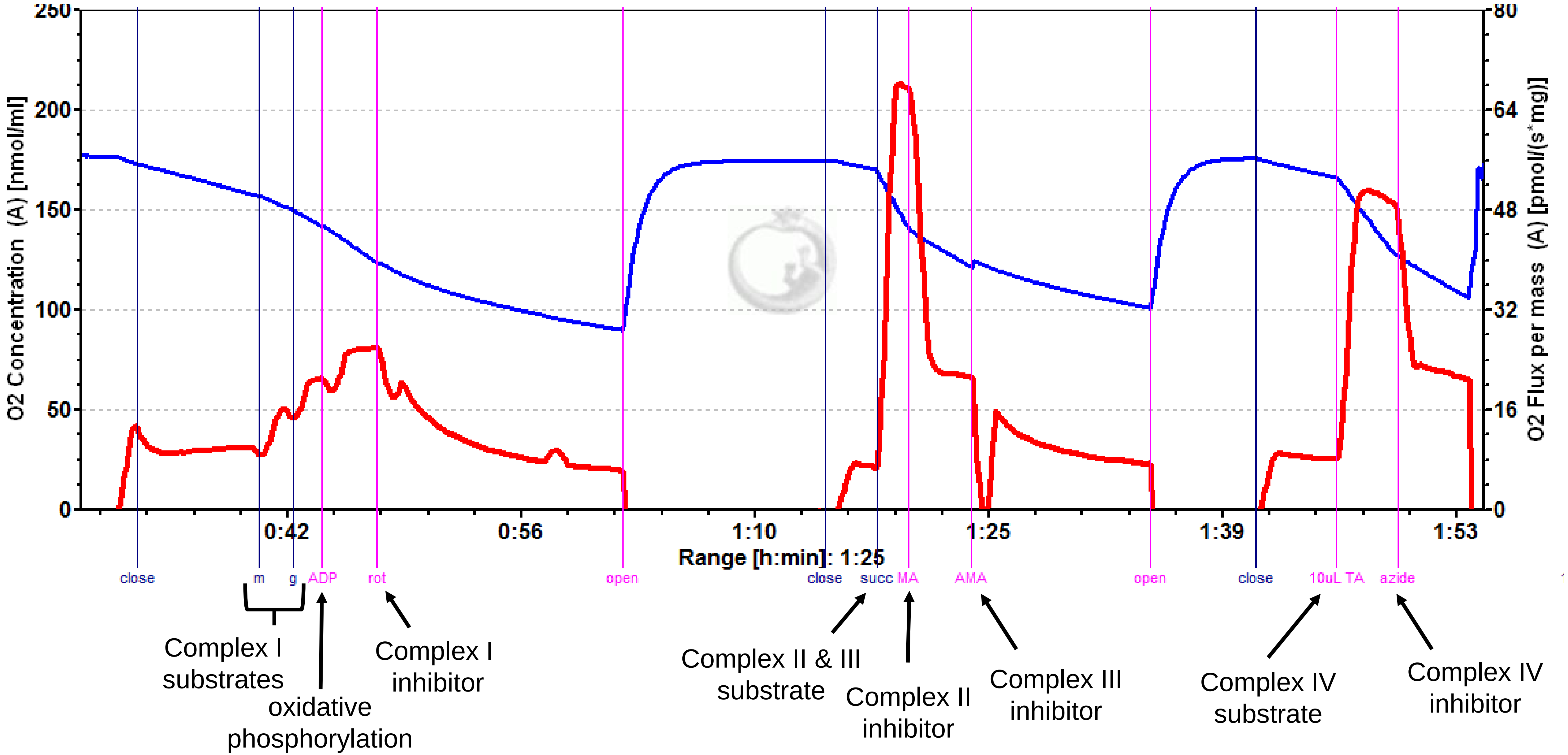

Same HRR trace for sham kidney
annotated to show calculated complex activity

Blue trace = oxygen concentration (nmol/mL)
Red trace = O2 flux (pmol / mg tissue / sec)

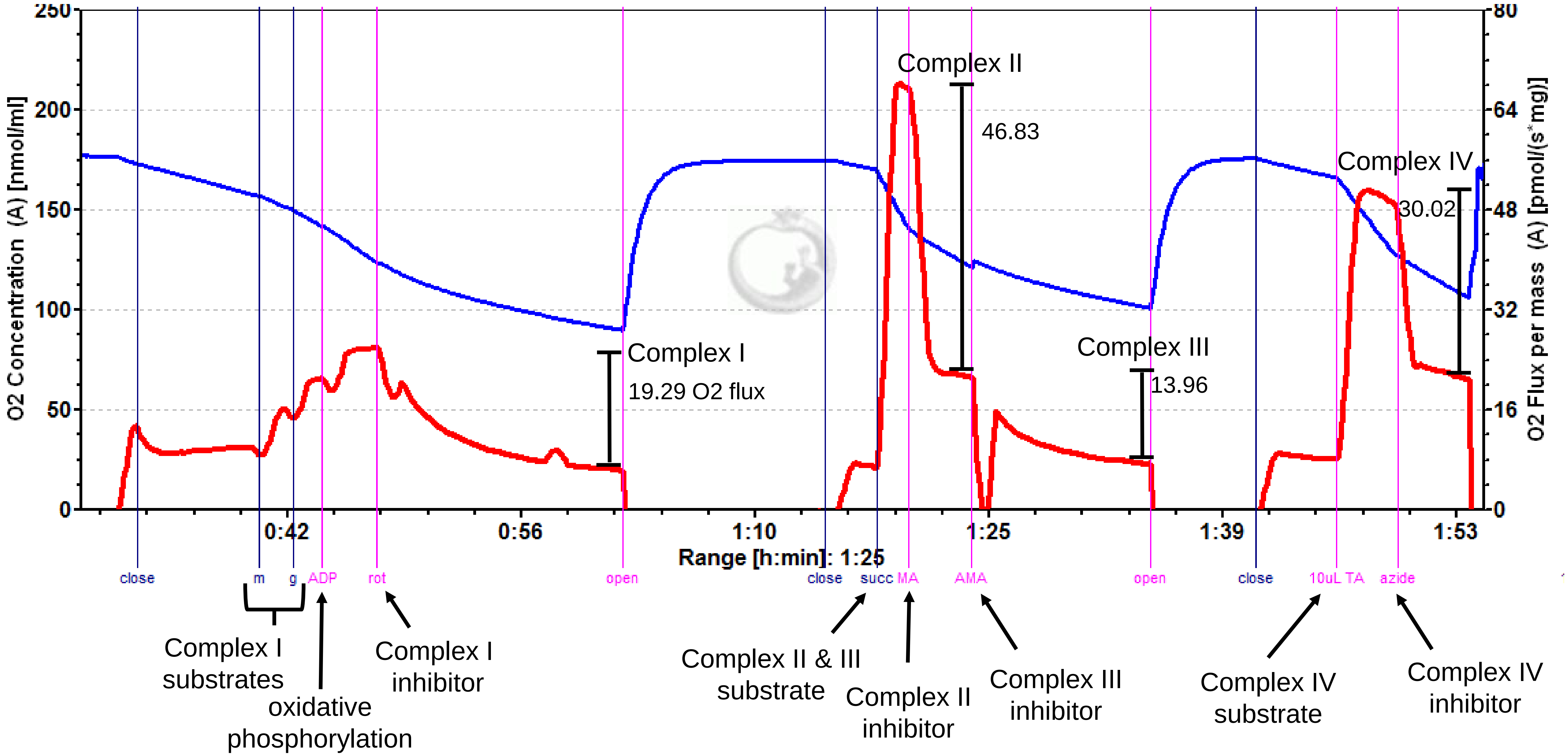

Abbreviations on previous slide	Substance used in HRR	Function
m/g	Malate / glutamate	Complex I substrate
ADP	Adenosine diphosphate	Oxidative phosphorylation
Rot	Rotenone	Complex I inhibitor
Succ	Succinate	Complex II & III substrate
MA	Malonic acid	Complex II inhibitor
AMA	Antimycin A	Complex III inhibitor
T/A	TMPD/Ascorbate	Complex IV substrate
Azide	Azide	Complex IV inhibitor

Complex activity is calculated by subtracting O2 flux of complex-specific substrates minus specific inhibitors, as follows:

Complex I respiration = O2 flux(ADP – Rot)
Complex II respiration = O2 flux(Succ – MA)
Complex III respiration = O2 flux(MA – AMA)
Complex IV respiration = O2 flux(T/A – azide)

For transparency, the following slide shows a representative IHC image of nitrotyrosine staining in cortical kidney tissue for each “n”

- **Figure 3A** is comprised of the representative image taken for Sham 2, CS/Tx 2, and CS/Tx+NS 3.

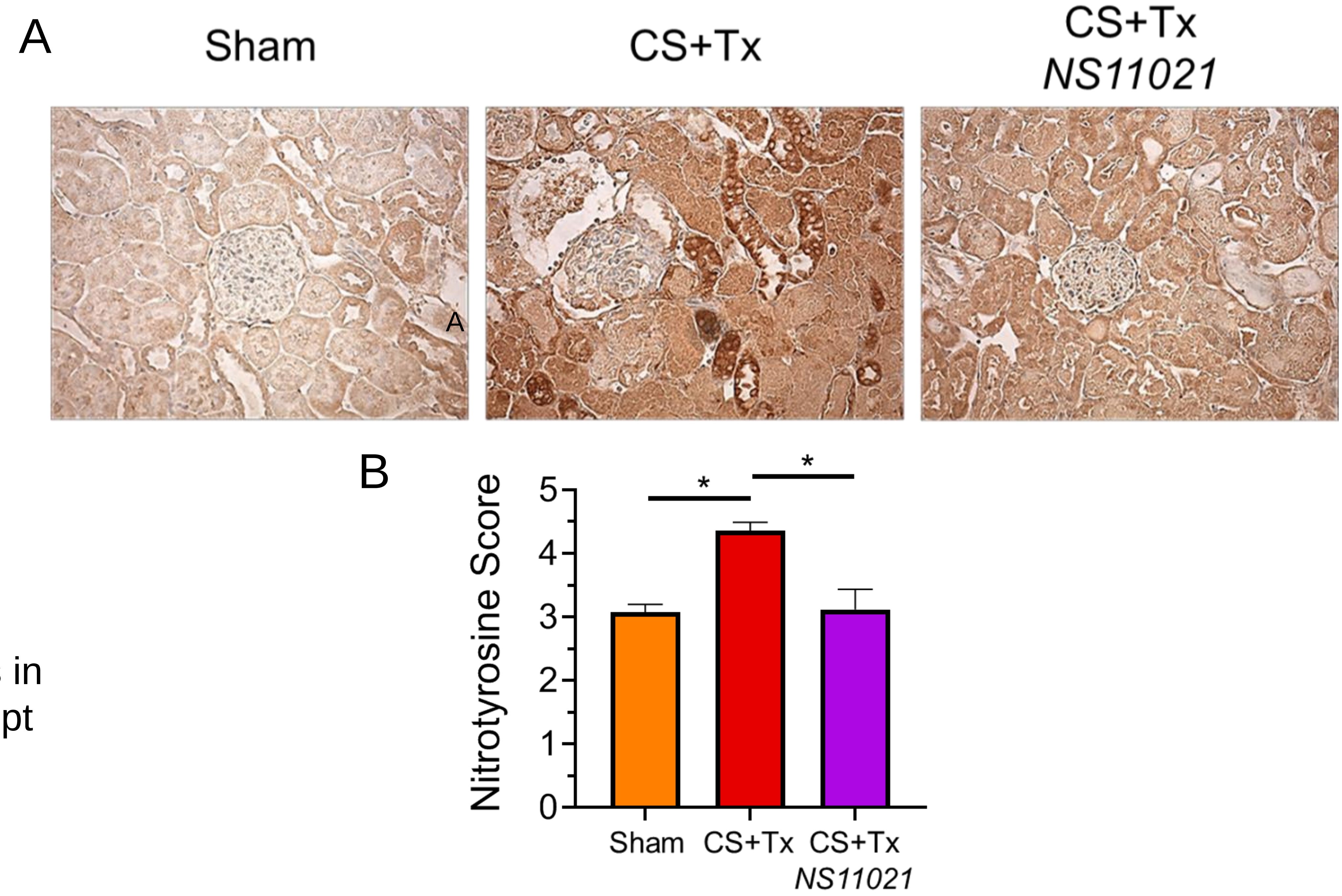

Figure 3
as it appears in
the manuscript

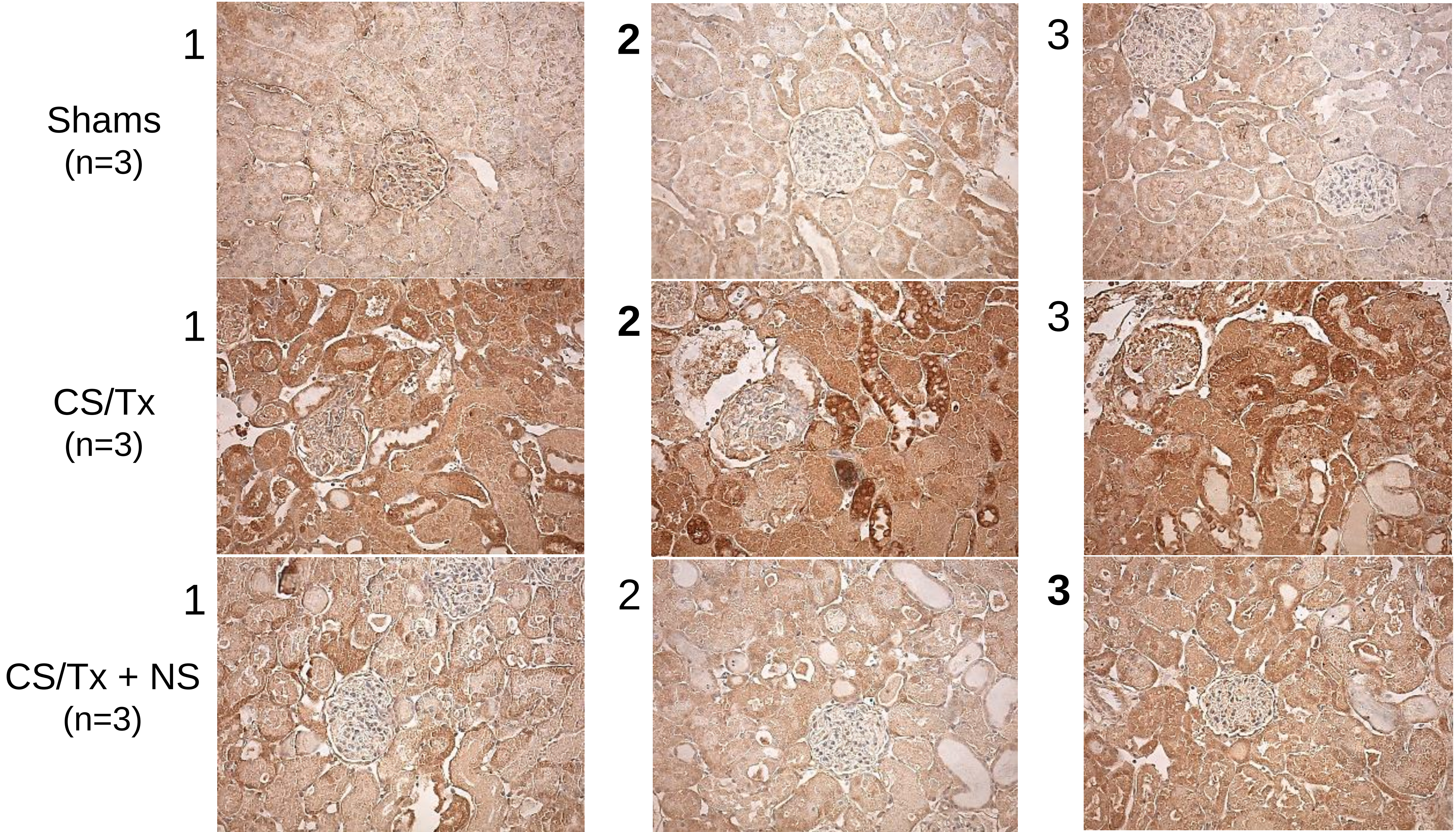
1
2
3
Shams
(n=3)
1
2
3
CS/Tx
(n=3)
1
2
3
CS/Tx + NS
(n=3)

The following slide shows a representative IHC image of TUNEL staining in cortical kidney tissue for each “n”

- **Figure 4A** is comprised of the representative image taken for Sham 1, CS/Tx 1, and CS/Tx+NS 1.

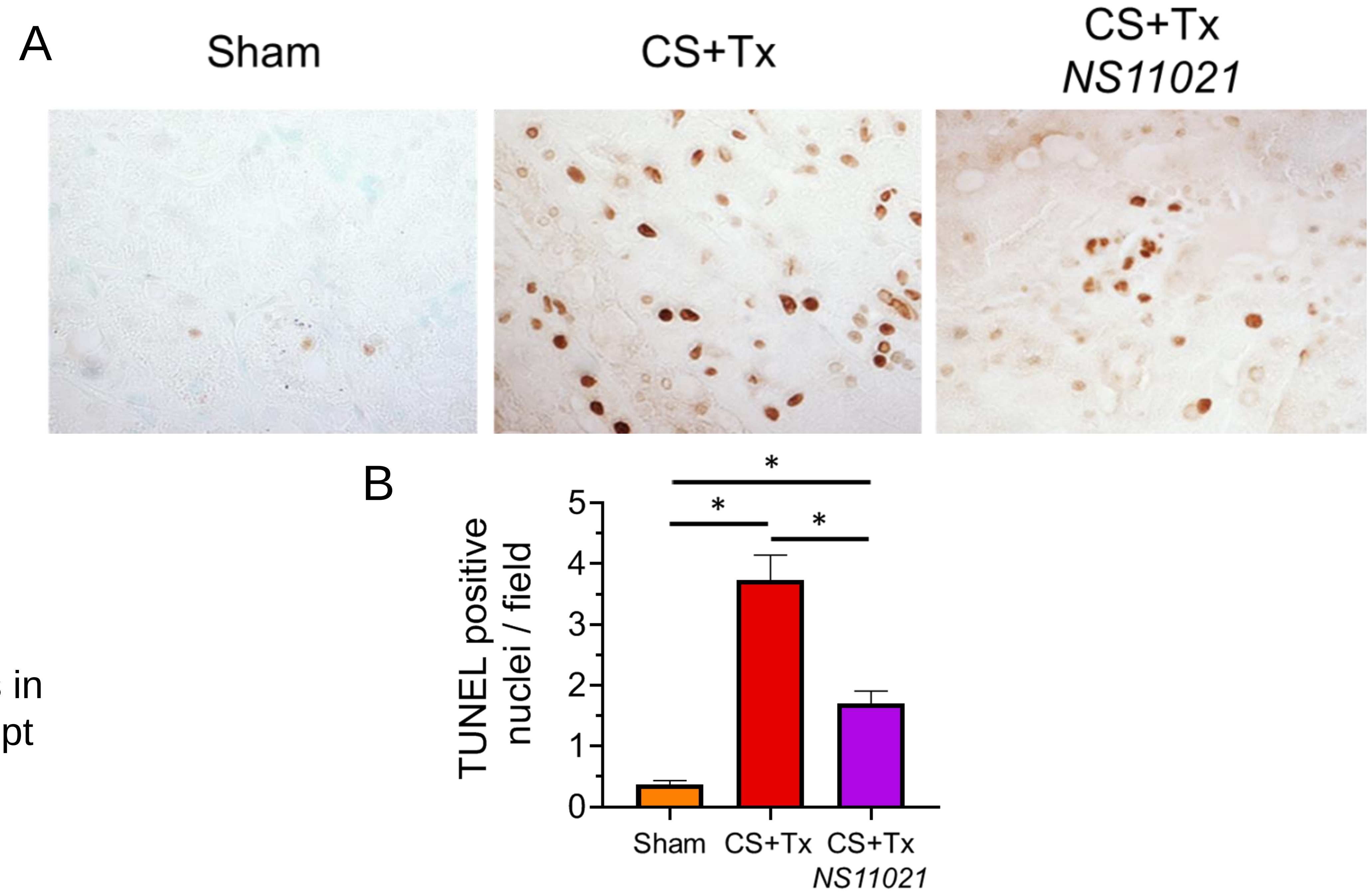

Figure 4
as it appears in
the manuscript

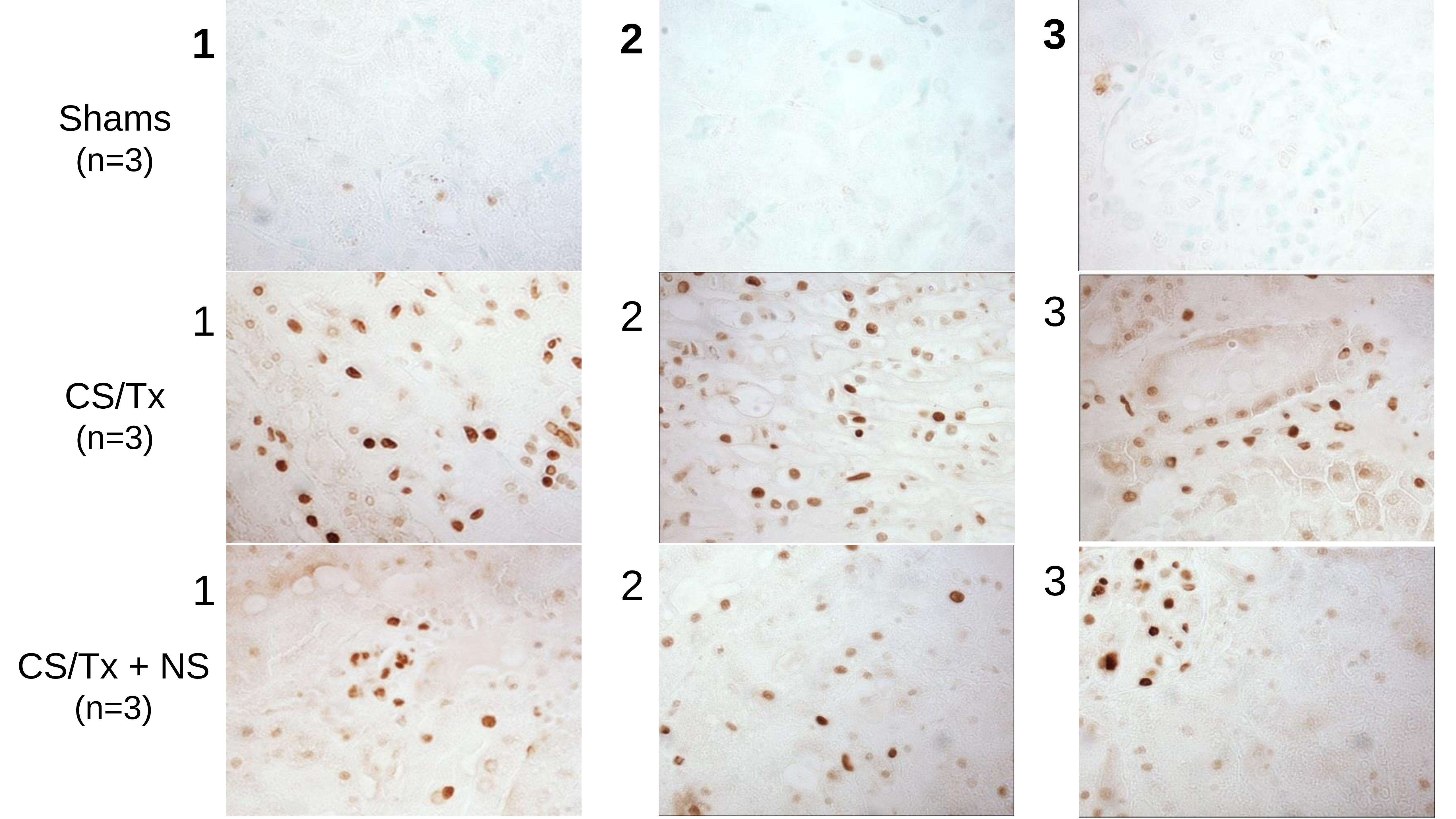

Shams
(n=3)
CS/Tx
(n=3)
CS/Tx + NS
(n=3)
1
2
3
1
2
3
1
2
3
